# A meta-analysis of resting-state fMRI in postherpetic neuralgia using AES-SDM

**DOI:** 10.3389/fnins.2025.1556639

**Published:** 2025-03-31

**Authors:** Guanzuan Wu, Yurou Luo, Danling Guo, Sangying Lv, Jianfeng Yang

**Affiliations:** ^1^Department of Radiology, Shaoxing People’s Hospital, Shaoxing, China; ^2^Department of Anesthesiology, Affiliated Hospital of Shaoxing University, Shaoxing, China

**Keywords:** postherpetic neuralgia, chronic pain, meta-analysis, AES-SDM, rs-fMRI

## Abstract

**Background:**

Resting-state fMRI (rs-fMRI) has revealed a range of neural activity patterns in patients with postherpetic neuralgia (PHN). However, inconsistencies in study design have led to conflicting findings in previous research studies. This meta-analysis used the anisotropic effect size-signed differential mapping (AES-SDM) approach to evaluate rs-fMRI studies on PHN and to provide more robust insights into the brain networks involved in processing PHN pain.

**Materials and methods:**

A systematic search of PubMed, Embase, Web of Science, and the Cochrane Database was performed for rs-fMRI studies comparing PHN patients with healthy controls, up until 1 November 2024. The AES-SDM approach was then employed to meta-analyze the abnormal brain activity patterns observed in PHN patients.

**Results:**

A total of eight articles were included in the analysis comprising 148 patients with PHN and 179 healthy controls. The meta-analysis found that patients with PHN exhibited increased activity in the right middle temporal gyrus (MTG.R), right precuneus (PCUN.R), and right superior frontal gyrus, orbital part (ORBsup.R). In contrast, a reduction in functional activity was observed in the left superior frontal gyrus, medial (SFGmed.L), left calcarine fissure/surrounding cortex (CAL.L), right precentral gyrus (PreCG.R), and right inferior temporal gyrus (ITG.R). Sensitivity analysis revealed that all of these regions exhibited high reproducibility, and no significant publication bias was identified.

**Conclusion:**

This meta-analysis reveals altered specific brain activity in PHN patients, providing a foundation for targeted treatments that address both sensory and affective aspects of chronic pain.

**Systematic review registration:**

PROSPERO, registration no. CRD42024614718; https://www.crd.york.ac.uk/PROSPERO/view/CRD42024614718.

## 1 Introduction

Postherpetic neuralgia (PHN) is a chronic neuropathic pain syndrome described as pain that lasts at least 90 days after the appearance of an acute herpes zoster rash (Johnson and Rice, 2014). The pain associated with PHN is often neuropathic, with patients reporting sensations such as burning, stabbing, or electrical shocks (Hadley et al., 2016). This condition is a significant cause of morbidity, with substantial impacts on quality of life, sleep, and emotional wellbeing (Mallick-Searle et al., 2016). The prevalence of PHN has steadily risen over the years, significantly exacerbating the societal medical burden (Thompson et al., 2021). Despite its high prevalence, the central nervous system mechanisms underlying PHN remain incompletely understood.

With the transition from gate control theory (Melzack and Wall, 1965) to “pain matrix” theory (Melzack, 1990), our understanding of pain has evolved from a simple sensory input to recognizing it as an output from a wide range of neural networks in the brain. Research on PHN also extends from herpes zoster infection, which exposes afflicted neurons to abnormal firing and inflammation, to central changes in the structure and function of the brain. Functional magnetic resonance imaging (fMRI), which uses fluctuations in blood oxygenation-level-dependent signals to examine changes in cerebral function, has emerged as an important tool for studying the brain’s role in pain processing (Biswal et al., 2010). According to a systematic review, PHN was found to be associated with brain functional abnormalities in the “pain matrix,” which includes the thalamus, insula, amygdala, parahippocampus, precentral gyrus, dorsolateral prefrontal cortex, and other areas such as the precuneus, lentiform, and brain stem (Tang et al., 2021). However, because of the limitations in imaging methods, scanning methods, sampling size, data analysis, and other methods, the results are heterogeneous, making it difficult to draw reliable and complete conclusions. Therefore, meta-analyses are necessary to analyze the previous findings.

Anisotropic effect size-signed differential mapping (AES-SDM) is an effective technique for delineating brain areas by coordinate-based meta-analysis of neuroimaging data (Radua and Mataix-Cols, 2009; Radua et al., 2012; Radua et al., 2014). In comparison to activation likelihood estimation (ALE), the AES-SDM method has higher sensitivity to small-effect regions, better ability to manage anisotropic voxel space, incorporates both positive and negative activation effects, and enhances the statistical management of heterogeneity across studies (Radua et al., 2012). This meta-analysis aimed to combine the findings of fMRI studies that examined the brain activity of patients with PHN using the AES-SDM. We aimed to identify consistent brain activation patterns associated with PHN and explore the possibility of alterations in pain processing circuitry by combining data from various analytical approaches, such as amplitude of low-frequency fluctuation (ALFF), fractional amplitude of low-frequency fluctuation (fALFF), regional homogeneity (ReHo), and functional connectivity density (FCD), among others. This analysis may help understand the neural mechanisms underlying PHN and inform the development of targeted therapeutic interventions for this complex condition.

## 2 Materials and methods

### 2.1 Search strategies

From inception to 1 November 2024, a systematic literature search was conducted across PubMed, Embase, Web of Science, and Cochrane Database, in accordance with the Preferred Reporting Items for Systematic Reviews and Meta-Analyses (PRISMA) guidelines (Page et al., 2021), and the results were registered with the International Prospective Register of Systematic Reviews at the University of York (PROSPERO, registration no. CRD42024614718). The search terms of PubMed were as follows: (“Neuralgia, Postherpetic” OR “postherpetic neuralgia” OR “PHN”) AND (“neuroimaging” OR “magnetic resonance imaging” OR “MRI” OR “amplitude of low-frequency fluctuation” OR “ALFF” OR “fractional amplitude of low-frequency fluctuation” OR “fALFF” OR “regional homogeneity” OR “ReHo” OR “independent component analysis” OR “functional connectivity”). The remaining three databases’ search methodologies were adjusted to fit with the aforementioned methodology. Moreover, review articles and the reference lists of the selected articles were examined for additional acceptable publications.

### 2.2 Inclusion and exclusion criteria

The inclusion criteria were as follows: (1) studies in which patients were selected based on PHN diagnostic criteria; (2) studies that used fMRI as the imaging technique; (3) studies that used whole-brain analysis to compare differences between PHN patients and healthy controls; (4) studies with results reported in standard stereotactic coordinate space (Talairach or the Montreal Neurological Institute, MNI); and (5) original article published in a peer-reviewed journal.

The exclusion criteria were as follows:(1) reviews, protocols, conference articles, letters, animal studies, or case reports; (2) patients who received any intervention or stimulation during or before the neuroimaging scan; and (3) studies that were conducted using seed-based functional connectivity analysis.

### 2.3 Data extraction and quality assessment

Two investigators independently analyzed the identified articles and subsequently examined the entire texts to determine compliance with the inclusion and exclusion criteria. The third author addressed and resolved any disagreements or uncertainties that arose between the two investigators. The following study information was gathered: (1) publication indexes; (2) demographic and clinical characteristics (sample size, sex, and mean age of PHN patients and healthy controls); (3) disease-associated variables (disease duration and VAS score); (4) neuroimaging method (scanning device and analytic approach); and (5) functional brain activity differences between PHN patients and healthy controls (peak coordinates of different regions in Talairach or MNI space, cluster size, and statistical threshold).

A 12-point checklist based on previous meta-analyses of fMRI studies was used to evaluate the quality of the included studies (Wang et al., 2024). The Quality Assessment Checklist included the demographical and clinical characteristics of the subjects, methods for image acquisition and analysis, and quality of results and conclusions. All included studies must meet the quality evaluation threshold, with a total score greater than 6.0.

### 2.4 Statistical analysis

The coordinate-based meta-analysis study was carried out using the AES-SDM (version 6.23, https://www.sdmproject.com/software). First, the peak coordinates and effect sizes (T-values) for the clusters of difference between PHN and healthy controls were gathered from each collection of data. The variance maps and effect sizes of the brain function differences were then reconstructed using an anisotropic Gaussian kernel with a full width at half maximum of the Gaussian kernel set to 20 mm (Radua et al., 2014). Thresholds (*p* < 0.005 with peak Z > 1 and a cluster extent >10 voxels) that optimize sensitivity and specificity are shown on the standardized template in MNI coordinates (Radua et al., 2010; Radua et al., 2012). The mean map was eventually produced by a voxel-wise calculation of the random effects mean of the study maps, which was weighted by the sample size, the variance of each study, and between-study heterogeneity. The Egger’s test was used to evaluate potential publication bias, with a threshold of *p* < 0.05 indicating statistical significance.

A jackknife sensitivity analysis was performed to evaluate the robustness of the findings by iteratively excluding one dataset at a time during the investigation (Radua and Mataix-Cols, 2009). If a brain region consistently shows significance across most or all study combinations, it can be concluded that this finding is highly reproducible.

Meta-regression analysis was also performed to explore potential correlations between mean age, VAS score, and disease duration in PHN patients. Consistent with previous meta-analyses, the potential influence of the aforementioned indicators is assessed through simple linear regression, weighted by the square root of the sample size, and limited to predicting feasible SDM values (i.e., from −1 to 1) within the observed range of the variable (Saarinen et al., 2020). To minimize false associations, the probability cutoff was restricted to *p* < 0.005. These procedures were only carried out in the regions recognized in the primary impact.

## 3 Results

### 3.1 Included studies and sample characteristics

**Figure 1:**
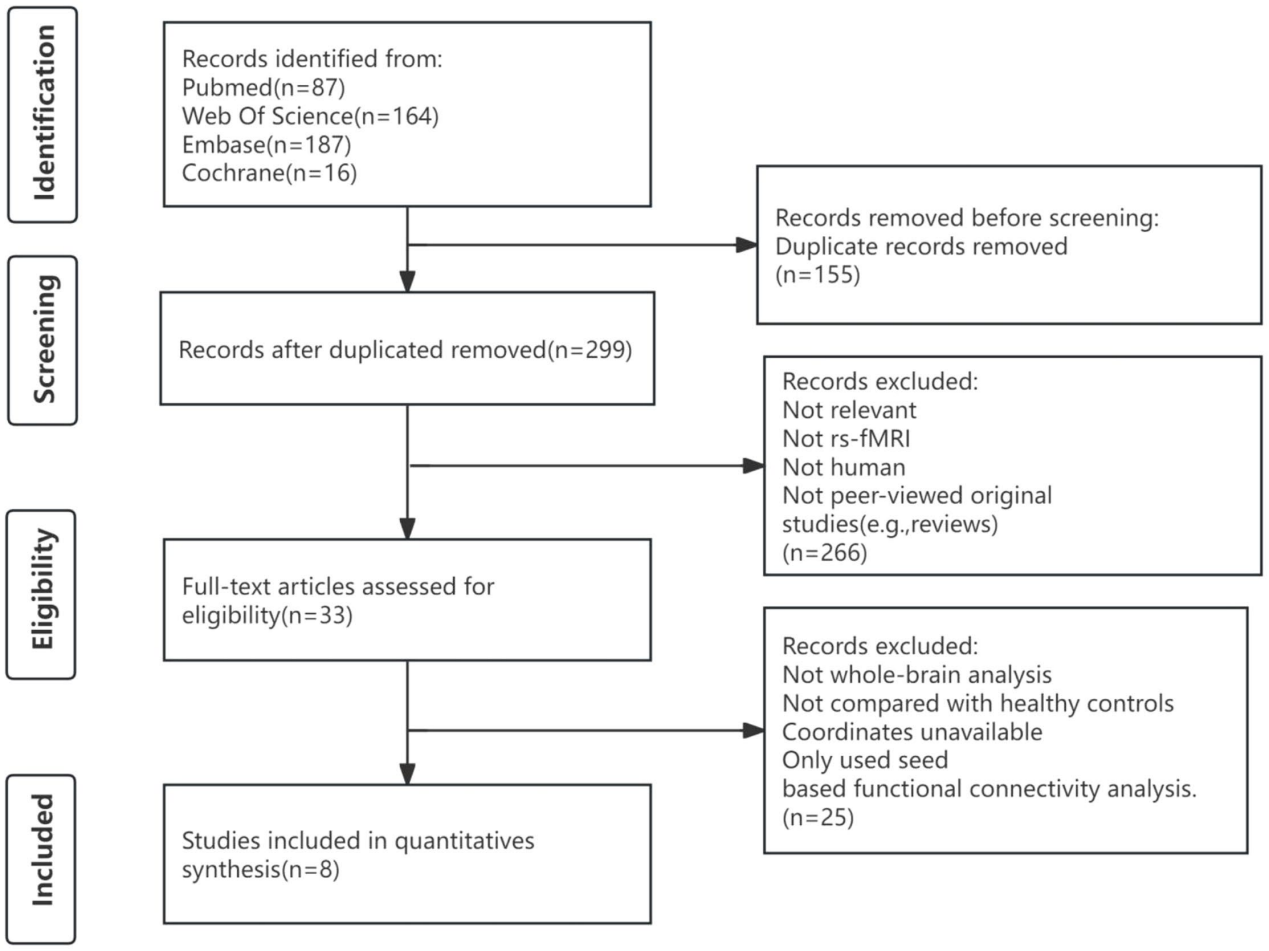
Flowchart to identify eligible studies for meta-analysis.

A total of 454 articles were retrieved using our search strategy, with 8 meeting the inclusion criteria for the meta-analysis. The flowchart shows the entire process (Figure 1). The reference lists of selected articles did not contain any relevant studies that met the criteria. The study used the following resting-state analysis methods: ALFF, fALFF, ReHo, and FCD. Three articles (Cao et al., 2017a; Cao et al., 2017b; Dai et al., 2020) employed two different analytical methods; as a result, the meta-analysis included 8 papers containing 11 studies (Xian et al., 2015; Cao et al., 2017a; Cao et al., 2017b; Hong et al., 2018; Gu et al., 2019; Dai et al., 2020; Huang et al., 2020; Jiang et al., 2024). This analysis involved148 PHN patients (78 male subjects and 70 female subjects) and 179 healthy controls (92 male subjects and 87 female subjects). Comprehensive information about the included research is shown in Table 1.

**Table 1:** Demographic and clinical characteristics of the studies included in the meta-analysis.

Study	Sample size (female)		Mean age (SD)		Disease duration (SD) (month)	VAS score	Analysis	Statistical threshold	Quality score
	Patients	HCs	Patients	HCs					
Cao et al. (2017a)	23(13)	55(21)	65.9 ± 2.3	63.1 ± 0.79	12.2 ± 3.7	6.7 ± 0.3	ReHofALFF	P_Alphasim_ < 0.05	11
Cao et al. (2017b)	19(8)	19(11)	64.4 ± 2.1	61.4 ± 1.1	5.4 ± 1.3	6.3 ± 0.4	ReHo fALFF	P_Alphasim_ < 0.05	11
Dai et al. (2020)	12(5)	12(5)	60.92 ± 9.29	61.75 ± 8.82	NA	NA	ALFF fALFF	P_Alphasim_ < 0.05	10.5
Gu et al. (2019)	18(7)	18(7)	59.67 ± 8.41	59.27 ± 7.74	3.89 ± 0.87	6.61 ± 1.47	ALFF	P_GRF_ < 0.05	11
Hong et al. (2018)	22(13)	28(19)	66.13 ± 6.77	54.21 ± 7.72	2.01 ± 1.37	7.36 ± 0.95	FCD	P_GRF_ < 0.05	11
Jiang et al. (2024)	22(11)	19(7)	63.1 ± 10.9	57.6 ± 7.3	2.85 ± 0.21	6.3 ± 1.0	FCD	P_GRF_ < 0.05	11
Huang et al. (2020)	24(9)	20(13)	67.0 ± 14.1	63.1 ± 12.2	7.57 ± 2.40	6.7 ± 1.6	ALFF	P_FDR_ < 0.05	11
Xian et al. (2015)	8(4)	8(4)	60.00 ± 7.01	NA	10.75 ± 5.52	5.88 ± 0.99	ALFF	P_Alphasim_ < 0.05	9.5

### 3.2 Meta-analysis results

**Figure 2:**
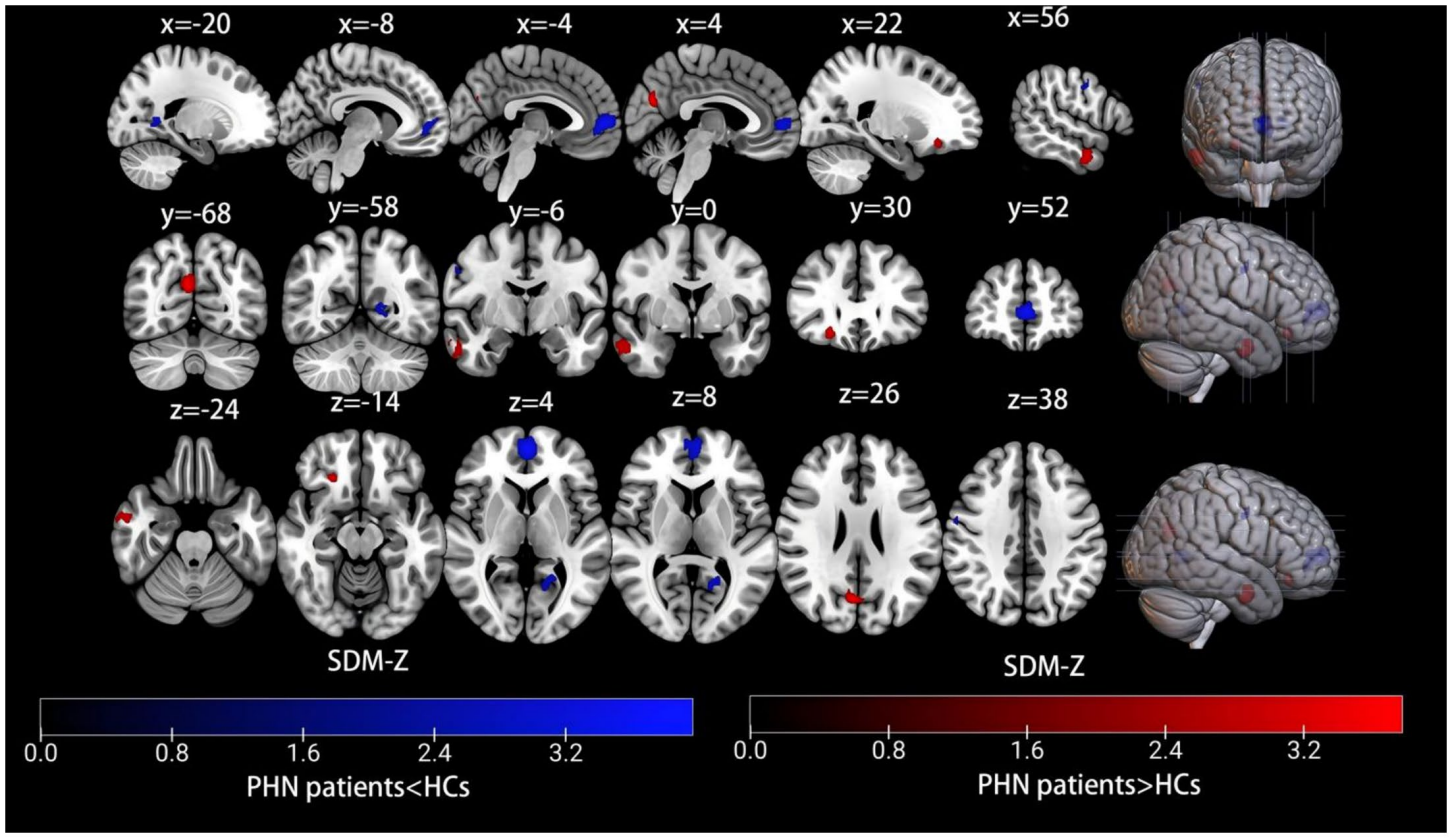
Meta-analysis comparing brain functional alterations between PHN patients and healthy controls. Red and blue clusters represent increased and decreased brain activity, respectively. The color bar indicates the maximum and minimum SDM-Z values. HCs, healthy controls; PHN, postherpetic neuralgia, SDM, seed-based mapping.

The AES-SDM meta-analysis results comparing PHN patients with healthy controls indicated a total of seven clusters, which are shown in Table 2 and Figure 2. Increased brain activity in PHN was mainly observed in the right middle temporal gyrus (MTG.R), right precuneus (PCUN.R), and right superior frontal gyrus, orbital part (ORBsup.R). In contrast, decreased brain activity in PHN was predominantly found in the left superior frontal gyrus, medial (SFGmed.L), left calcarine fissure/surrounding cortex (CAL.L), right precentral gyrus (PreCG.R), and right inferior temporal gyrus (ITG.R).

**Table 2:** Clusters of voxels with abnormal brain activity in PHN patients compared to healthy controls.

Anatomical label	peak MNI coordinates (x, y, z)	Cluster size^a^	SDM-Z value^b^	*p*-value^c^	Jackknife sensitivity analysis	Egger’s test (bias test)	Cluster breakdown (No. of voxels)
PHN > HCs							
R middle temporal gyrus, BA 21	56,-2,-26	147	3.406	0.00033	9/11	Bias:0.21 Z:0.12 P:0.90	R middle temporal gyrus, BA 21(103) R inferior temporal gyrus, BA 21(27) R inferior temporal gyrus, BA 20(11) R middle temporal gyrus, BA 20(4) R inferior network, inferior longitudinal fasciculus(2)
R precuneus	6,-68,26	132	3.815	0.00007	10/11	Bias:-0.04 Z:-0.02 P:0.982	R precuneus (34) L cuneus cortex (32) R cuneus cortex (23) R precuneus, BA 23 (15) R cuneus cortex, BA 23(9) L precuneus(8) R median network, cingulum(6) L calcarine fissure / surrounding cortex(1) Corpus callosum(1) L calcarine fissure / surrounding cortex, BA 23(1) R cuneus cortex, BA 18(1) (undefined)(1)
R superior frontal gyrus, orbital part, BA 11	22,30,-14	42	3.517	0.00022	8/11	Bias:0.29 Z:0.17 P:0.866	R superior frontal gyrus, orbital part, BA 11(17) R inferior frontal gyrus, orbital part, BA 11(10) R frontal orbito-polar tract(6) R inferior frontal gyrus, orbital part, BA 47(1) R middle frontal gyrus, orbital part, BA 11(1) (undefined), BA 11(7)
PHN < HCs							
L superior frontal gyrus, medial	0,54,2	416	−4.014	0.00003	9/11	Bias:-0.41 Z:-0.24 P:0.812	L superior frontal gyrus, medial, BA 10(118) L anterior cingulate / paracingulate gyri, BA 10(102) R superior frontal gyrus, medial, BA 10(86) L superior frontal gyrus, medial(27) L superior frontal gyrus, medial orbital, BA 10(22) L anterior cingulate / paracingulate gyri, BA 32(15) R superior frontal gyrus, medial(12) R superior frontal gyrus, medial orbital, BA 10(7) L superior frontal gyrus, medial orbital(7) R superior frontal gyrus, medial orbital(5) L anterior cingulate / paracingulate gyri(4) R anterior cingulate / paracingulate gyri, BA 10(1) L superior frontal gyrus, medial, BA 32(1) R anterior cingulate / paracingulate gyri(1) (undefined), BA 10(5) (undefined)(3)
L calcarine fissure / surrounding cortex, BA 19	−20,-52,6	93	−3.266	0.00055	8/11	Bias:-0.36 Z:-0.22 P:0.827	L calcarine fissure / surrounding cortex, BA 17(32) Corpus callosum(29) L inferior network, inferior longitudinal fasciculus(9) L precuneus, BA 19(8) L calcarine fissure / surrounding cortex, BA 19(5) (undefined)(10)
R precentral gyrus, BA 6	56,-2,44	24	−2.844	0.00223	8/11	Bias:-0.28 Z:-0.17 P:0.865	R postcentral gyrus, BA 4(9) R precentral gyrus, BA 4(7) R precentral gyrus, BA 6(6) R postcentral gyrus, BA 3(2)
R inferior temporal gyrus, BA 20	56,-30,-24	19	−2.936	0.00166	8/11	Bias:-0.25 Z:-0.15 P:0.880	R inferior temporal gyrus, BA 20(19)

R, right; L, left; MNI, montreal neurological institute; BA, brodmann area; SDM, seed-based d mapping; PHN, postherpetic neuralgia; HCs, healthy controls.

a Cluster extent threshold: regions with <10 voxels are not reported in the cluster breakdown.

b Peak height threshold: *z* > 1.

c Voxel probability threshold: *p* < 0.005.

The results of Egger’s test for the MTG.R (Bias 0.21, Z 0.12, P 0.90), PCUN.R (Bias −0.04, Z -0.02, P 0.982), ORBsup.R (Bias 0.29, Z 0.17, P 0.866), SFGmed.L (Bias −0.41, Z -0.24, P 0.812), CAL.L (Bias −0.36, Z -0.22, P 0.827), PreCG.R (Bias −0.28, Z -0.17, P 0.865), and ITG.R (Bias −0.25, Z -0.15, P 0.880) confirmed no significant heterogeneity among the studies (Table 2). A whole-brain analysis of the sensitivity of the jackknife revealed high reproducibility in all brain regions. The results for the PCUN.R were replicated in 10 out of 11 iterations, while the MTG.R and SFGmed.L were replicated in 9 out of 11 iterations. The ORBsup.R, CAL.L, SFGmed.L, and ITG.R were replicated in 8 out of 11 iterations (Table 3).

**Table 3:** Results of sensitive analysis.

Discarded article	Analysis	Increased			Decreased			
		R MTG	R PCUN	R ORBsup	L SFGmed	L CAL	R PreCG	R ITG
Cao et al. (2017a)	ReHo	Yes	Yes	No	Yes	Yes	Yes	No
Cao et al. (2017a)	fALFF	Yes	Yes	Yes	Yes	No	Yes	Yes
Cao et al. (2017b)	ReHo	Yes	Yes	Yes	Yes	Yes	No	No
Cao et al. (2017b)	fALFF	Yes	Yes	No	No	No	Yes	Yes
Dai et al. (2020)	ALFF	No	Yes	Yes	Yes	Yes	Yes	Yes
Dai et al. (2020)	fALFF	Yes	Yes	Yes	Yes	Yes	Yes	Yes
Gu et al. (2019)	ALFF	Yes	Yes	Yes	Yes	Yes	Yes	Yes
Hong et al. (2018)	FCD	Yes	Yes	Yes	Yes	Yes	Yes	Yes
Jiang et al. (2024)	FCD	Yes	Yes	Yes	No	Yes	No	Yes
Huang et al. (2020)	ALFF	No	No	Yes	Yes	Yes	Yes	Yes
Xian et al. (2015)	ALFF	Yes	Yes	No	Yes	No	No	No
		9/11	10/11	8/11	9/11	8/11	8/11	8/11

R, right; L, left; MTG, middle temporal gyrus; PCUN, precuneus; ORBsup, superior frontal gyrus, orbital part; SFGmed, superior frontal gyrus, medial; CAL, calcarine fissure/ surrounding cortex; PreCG, precentral gyrus; ITG, inferior temporal gyrus.

A meta-regression analysis was conducted to investigate the potential relationship between mean age, VAS score, and disease duration in patients with PHN. The findings indicated that the left precuneus (PCUN.L) exhibited a positive correlation with mean age and disease duration. Additionally, the left inferior network and inferior longitudinal fasciculus was positively correlated with the VAS score (Table 4).

**Table 4:** Correlation between alterations in brain activity and age, disease duration, and VAS score in PHN revealed by meta-regression analysis.

Factor	Anatomical label	peak MNI coordinates (x, y, z)	Cluster size^a^	SDM-Z value^b^	p-value^c^
Age	L precuneus, BA 7	−4,-72,46	140	3.588	0.00017
Disease duration	L precuneus, BA 7	0,-74,42	94	3.262	0.00055
VAS score	L inferior network, inferior longitudinal fasciculus	−38,-44,-14	14	3.444	0.00029

a Cluster extent threshold: regions with <10 voxels are not reported in the cluster breakdown.

b Peak height threshold: *z* > 1.

c Voxel probability threshold: *p* < 0.005.

L, left; MNI, Montreal Neurological Institute; BA, Brodmann area; SDM, seed-based mapping.

## 4 Discussion

This was the initial rs-fMRI meta-analysis using AES-SDM to investigate changes in brain activity in PHN patients. The analysis revealed increased brain activity in the MTG.R, PCUN.R, and ORBsup.R, while decreased brain activity was found in the SFGmed.L, CAL.L, PreCG.R, and ITG.R. The sensitivity of the jackknife analysis revealed high reproducibility in all brain regions. Furthermore, meta-regression analysis indicated that the PCUN.L exhibited a positive correlation with mean age and disease duration, whereas the left inferior network and inferior longitudinal fasciculus had a positive correlation with the VAS score.

Pain is a multidimensional experience that includes sensory-discriminative, affective-motivational, and cognitive-evaluative components (Mercer Lindsay et al., 2021). Neuroimaging and neurophysiological studies on humans have shown that noxious stimuli consistently activate a network of brain areas; these brain areas, or a subset or superset of them, have been termed the “pain matrix” and are used as a brain signature for a pattern of activity associated with pain (Legrain et al., 2011). In contrast to acute pain, neuroimaging studies on chronic pain involve subgroup comparisons between chronic pain patients and healthy volunteers. Recent evidence has refuted the “pain matrix” hypothesis by challenging the notion that there is no uniform set of brain regions that can be equated with the presence of pain, particularly for different clinical chronic pain conditions that show unique patterns of brain activity (Apkarian et al., 2011). This meta-analysis reveals distinct brain activity patterns in PHN, differentiating it from other chronic pain disorders such as cervical spondylosis (Zhang and Ding, 2024) and knee osteoarthritis (Cheng et al., 2024).

The regions that showed the most significant decline in brain activity were the SFGmed, CAL, PreCG, and ITG, with more pronounced decrease observed in the SFGmed. This suggests that the abnormal activity patterns in the SFGmed may be a crucial central pathological feature of PHN. Functional deactivation in PHN pain patients is consistent with findings from other chronic pain patients. The review indicates that activation of the SFGmed occurs during the perception of acute pain stimuli, while chronic pain patients typically show functional deactivation in this region (Seminowicz and Moayedi, 2017). The SFGmed is a major node of the default mode network (DMN) that is anatomically connected to the descending pain modulatory system (Menon, 2023). This region is important for processing both the affective and cognitive components of pain sensations (Bushnell et al., 2013). The PCUN is also a part of the DMN and, as found in our meta-analysis, has shown significantly increased functional activity. Additionally, it was positively correlated with both mean age and disease duration. Due to a lack of localized lesions in this deeply seated region and a limited understanding of its architecture, the precuneus’ role in complex cognitive activities has remained relatively unclear (Dadario and Sughrue, 2023). A recent MRS study also found higher levels of GABA+/tCr in the PCUN of PHN patients (Wu et al., 2022a). Alterations in the SFGmed and PCUN within the DMN suggest that this network may exhibit a condition of sustained hyperexcitability in PHN. Previous studies (Wu et al., 2020; Wu et al., 2022b; Wu et al., 2025) have found that the abnormal functional connectivity of the DMN in PHN patients includes decreased internal connectivity, abnormal cross-network connectivity, and impaired dynamic connectivity. These changes may contribute to the persistence of pain, emotional disturbances, and cognitive dysfunction.

Changes in brain function activity were found in both the MTG and ITG, with the MTG showing the most significant activation in brain function activity. Previously, the MTG and ITG were not considered to play a role in pain processing, which includes language and semantic memory processing, visual perception, and multimodal sensory integration (Onitsuka et al., 2004). However, some recent studies have found structural and functional alterations in the temporal cortex of patients with various chronic pain disorders (Niddam et al., 2017; Chehadi et al., 2018; Henssen et al., 2019; Liu et al., 2023). The functions of the MTG and ITG in chronic pain may be related to higher cognitive functions, such as memory and emotion processing (Houde et al., 2020). Chronic pain frequently impacts the mood, behavior, and overall quality of life in PHN patients. The MTG and ITG may serve as potential targets for mood therapy.

The ORBsup is an important part of the reward system (Farr et al., 2016) and has been shown to have increased functional activity in our study. Fundamentally, pain is an unpleasant experience, and alleviating aversive states, including pain, can produce rewarding effects (Navratilova and Porreca, 2014). The activation of the ORBsup could indicate an adaptive change designed to counteract the negative effects of pain. Recent studies (Becker et al., 2017) have found the brain circuit that suppresses pain when a reward is present, and the inhibitory effect of reward on pain is mediated by the orbitofrontal cortex, which has a positive correlation with pain. The PreCG is known as the primary motor area (Banker and Tadi, 2023). It has been shown to have reduced functional activity in PHN. If the pain caused by PHN is severe, the brain may change its motor plan to avoid moving the painful area. This can result in movement inhibition and a reduced range of motion, which can eventually lead to muscle atrophy or joint dysfunction (Butera et al., 2016). The CAL, located in the occipital lobe of the brain, is involved in visuospatial attention, which refers to the ability to focus on visual stimuli and orient attention to specific locations in the visual field (Wandell et al., 2009). Chronic pain patients frequently have a heightened focus on the painful area of the body, which can lead to increased attentional processing of the affected region. This enhanced attentional bias toward the pain site may result in increased pain perception (Cheng et al., 2024).

Some limitations in this study need to be taken into account. First, to fully describe the changes in brain activity in PHN, this meta-analysis combines different methods of analysis, such as ALFF, fALFF, ReHo, and FCD, which may increase the heterogeneity of the study but may also provide complementary results. Eventually, different analytical methods produced similar results, and we proved the robustness of our findings using the jackknife sensitivity analysis. Second, although this meta-analysis included as many PHN rs-fMRI studies with different analysis methods as possible, the number of rs-fMRI studies was still limited, and the total sample size of the studies was small. Third, only articles published in English that used a global brain analysis were included in the study. The majority of the studies were conducted in China, and the studies published in other languages, as well as unpublished information, were excluded, as these factors may potentially introduce bias into the results. Finally, this meta-analysis only included cross-sectional studies; future longitudinal studies can help us understand the central neural system mechanisms underlying the transition from acute to chronic pain.

## 5 Conclusion

This meta-analysis reveals altered specific brain activity in PHN patients. Alterations in SFGmed and PCUN within the DMN indicate that this network may exhibit a condition of sustained hyperexcitability in PHN. Moreover, aberrant activity in MTG, ITG, ORBsup, PreCG, and CAL affects emotion regulation, memory processing, reward circuitry, and motor control. The observed changes in the DMN may reflect the interplay between chronic pain and self-referential processing, while disturbances in emotional and reward-related regions may contribute to the affective and motivational components of chronic pain. Alterations in motor control regions may indicate compensatory or maladaptive mechanisms related to the physical manifestation of pain. These findings provide a basis for targeted treatments that address both the sensory and affective aspects of chronic pain.

## Data Availability

The original contributions presented in the study are included in the article/supplementary material, further inquiries can be directed to the corresponding author.
